# Changes in an Arbuscular Mycorrhizal Fungi Community Along an Environmental Gradient

**DOI:** 10.3390/plants9010052

**Published:** 2020-01-01

**Authors:** Larissa Cardoso Vieira, Danielle Karla Alves da Silva, Indra Elena Costa Escobar, Julyana Maria da Silva, Ingrid Andrêssa de Moura, Fritz Oehl, Gladstone Alves da Silva

**Affiliations:** 1 Programa de Pós-Graduação em Biologia de Fungos, Departamento de Micologia, Universidade Federal de Pernambuco, Avenida da Engenharia, s/n, Recife, PE 50740-600, Brazil; indraescobar@gmail.com (I.E.C.E.); gladstonesilva@yahoo.com (G.A.d.S.); 2 Programa de Pós-Graduação em Ecologia e Monitoramento Ambiental, Departamento de Engenharia e Meio Ambiente, Universidade Federal da Paraíba, Av. Santa Elisabete, s/n, Centro, Rio Tinto, PB 58297-000, Brazil; daniellekarlas@yahoo.com.br; 3 Departamento de Micologia, Universidade Federal de Pernambuco, Avenida da Engenharia, s/n, Recife, PE 50740-600, Brazil; jumariaufpe@gmail.com (J.M.d.S.); ingridandressa.31@gmail.com (I.A.d.M.); 4 Agroscope, Competence Division for Plants and Plant Products, Ecotoxicology, Müller-Thurgau-Strasse 29, CH-8820 Wädenswil, Switzerland; fritz.oehl@gmail.com

**Keywords:** dry forests, Glomeromycota, edaphic properties, Tropics

## Abstract

Gradual environmental changes are determining factors in the disposition of plants and associated organisms, such as arbuscular mycorrhizal fungi (AMF). The objective of this study was to evaluate the AMF species communities in a tropical semi-arid region of NE Brazil under decreasing clay content at a mountain top area forming a vegetative mosaic of dry forests, savanna-like shrubland and humid montane forests. Through field and trap culture samples, 80 species of AMF were identified belonging to 25 genera, of which *Acaulospora* and *Glomus* were the most representative. In general, representatives of the order Gigasporales were indicators of sites with lower clay content and showed greater abundance in these sites. As expected, less richness was found in the site with higher clay content, but there was no variation in the Shannon-Weaver index in the gradient studied. The areas showed different assemblies of AMF among the sites with higher and lower clay content, and the main factors structuring the species were carbon, clay and potential acidity. In addition, field samples and trap cultures showed different assemblies; through the use of cultures it was possible to detect additional species. Soil properties have been found to be determinants for the distribution of these microorganisms and further studies in different vegetation types can help to understand the ecological preferences of AMF species.

## 1. Introduction

Arbuscular mycorrhizal fungi (AMF) are present in the soil in the form of spores and extraradicular hyphae, as well as in the roots of plants with which they form a mutualistic association. AMF receive carbohydrates and lipids necessary for their survival through the plants, in exchange they increase the availability of water and nutrients for the host [1,2]. Fungi obtain these resources for plants with greater efficiency, leading to an increase in plant productivity [3]. AMF are also important for soil because they promote the formation of aggregates through the action of extraradicular mycelia [4], and are able to improve soil structure by stabilizing the particles into larger and more stable structures [5]. Thus, they are relevant for agricultural areas and natural ecosystems, performing important functions for soil conservation by reducing erosion, recovering degraded areas and storing carbon in the soil [6].

The composition of AMF communities can be influenced by different biotic and abiotic factors, which act as filters for species selection [7]. Thus, knowledge of AMF diversity enables the understanding of the ecological preferences of these organisms at each site. Given the tight interaction between the plant and the fungus, the composition of the plant community may be associated with the structure of the AMF community [8]. In addition to this influence exerted by plant hosts, soil properties have been found to affect the AMF community [9], especially in terms of the availability of nutrients [5] and variations in pH [10,11].

Soil is composed of different types of particles (liquid, solid and gaseous), where the solid fraction is formed of various sizes and distributed among sand, silt and clay, of which sand constitutes the largest particle size, while clay represents the smallest dimension [12]. Soil structure consists of the arrangement of these particles in micro-aggregates that are connected by soil organisms and the substances they produce, forming larger aggregates [13]. Among the soil aggregates, porous spaces are formed that retain water and air to varying amounts depending on the type of particle; in general, clayey soils have greater water retention capacity than sandy soils [14]. Size and spacing among pores determine how much water and nutritional resources will be available for the plant roots and soil microorganisms (e.g., AMF) [15], as well as the distribution and activity of these organisms.

Different soil types influence AMF communities [16,17]. Soil texture affects the diversity of AMF [18] and the types of propagules according to the taxonomic group [19]. Gigasporaceae species have more robust hyphae [20] and higher mycelial growth in sandy soils; on the other hand, Glomeraceae species are more prevalent in soils with higher clay content [19], which may represent adaptation of species to these soil conditions. The size of the soil particles can change the distribution and expansion of hyphae of AMF, mycelial growth being higher in soils of coarse (sandy) particles in relation to those of fine particles [21]. In addition, sandy soils can stimulate mycorrhizal colonization by favoring root growth due to greater porosity, while clay soil is less porous and restricts root growth [22].

In light of the above, we expect to find differences in AMF species across textural changes in soil. The objective of this work was to understand the distribution of the AMF assembly in soils with a clay gradient in a mountain top area forming a vegetative mosaic of savanna-like shrublands, dry forests and humid montane forests within the semi-arid-Caatinga biome in NE Brazil. We identified AMF spores directly from field soils and additionally, for a more accurate species identification, also from trap cultures in the greenhouse. We hypothesized that the AMF species richness and diversity might be lower in soils with higher clay content, due to the smaller number of pores available for the propagation of AMF, minimizing the chances of propagation on host roots and consequent reduced spore production.

## 2. Results

### 2.1. Soil Properties

A gradual increase in the clay content from LCC to HCC was observed, and all areas differ significantly for this soil attribute (Table 1). A higher percentage of sand was found in LCC, and the silt content was higher in MCC and LCC. All three areas have acid pH with the lowest values in LCC, which differed significantly from the other areas in also having lower contents of potassium, magnesium and calcium, and a higher amount of aluminum and potential acidity (Table 2). The amount of phosphorus was highest in HCC, but differed significantly only from MCC. The TOC content was higher in MCC and LCC, differing statistically from HCC. A similar result was observed for humidity, which was higher in LCC and MCC (12.9% and 11.7%, respectively) than in HCC (7.2%). On the other hand, the electrical conductivity was highest in HCC. There was no difference in the amount of sodium among environments.

### 2.2. AMF Richness in the Field

**Figure 1:**
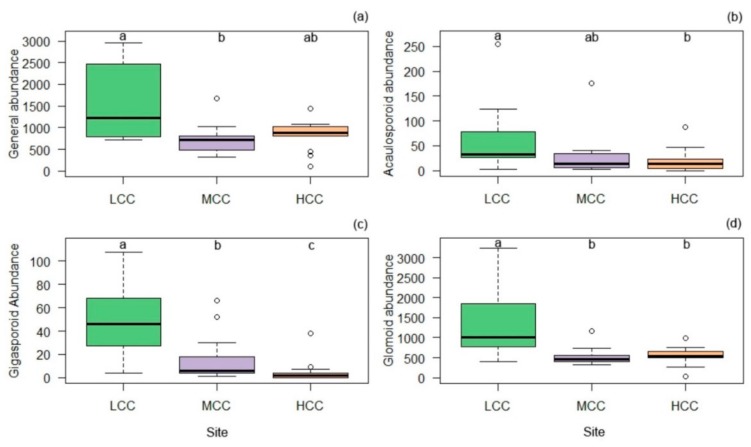
Overall number of spores (**a**) and by spore-formation type: acaulosporoid (**b**), gigasporoid (**c**) and glomoid (**d**) soil in three areas with clay gradient (LCC: Low Clay Content, MCC: Medium Clay Content, HCC: High Clay Content). Bars followed by the same letter do not differ statistically.

In general, spore density was highest in LCC, but this differed significantly only from MCC (Figure 1). The acaulosporoid type of spore formation was more abundant in LCC and MCC, while the gigasporoid and glomoid formations predominated only in LCC (Figure 1). The gigasporoid type of spore formation is an indicator of the LCC and MCC areas (Table 3).

A total of 77 AMF species were identified, distributed in 24 genera and 13 families (Table 4), of which 50 taxa were identified in the LCC area, 55 in MCC and 42 in the HCC area. The richness per sample differed among the areas (F = 4.11, *p* < 0.05), being lowest in HCC (13.2), and differing significantly from MCC (16.73) and LCC (16.8). The predominant genera at the study site were *Acaulospora*, with 20 species, followed by *Glomus* with 14 taxa.

**Figure 2:**
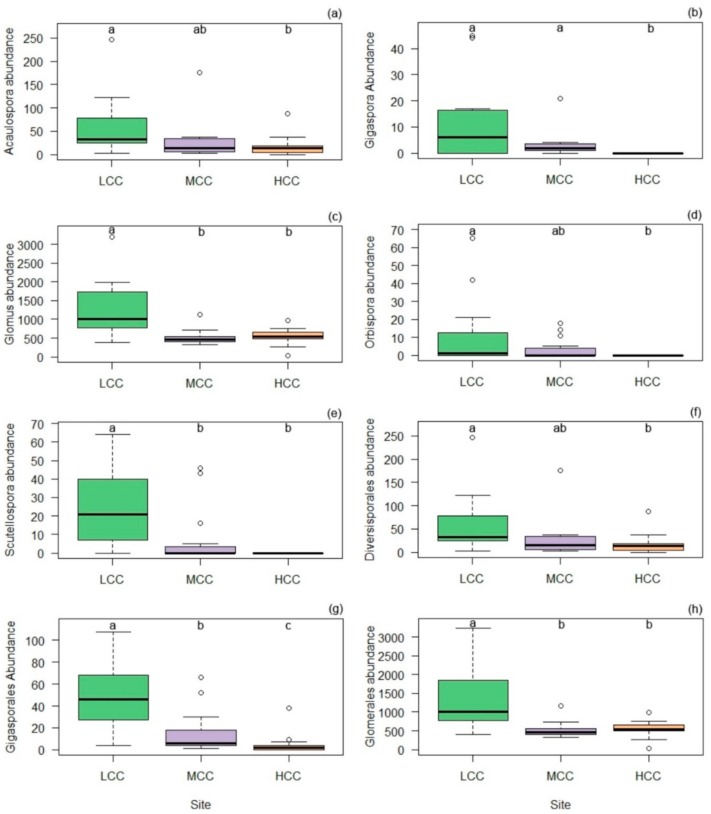
Abundance of genera (**a**–**e**) and orders (**f**–**h**) of AMF in soil in three areas with a clay gradient (LCC: Low Clay Content, MCC: Medium Clay Content, HCC: High Clay Content). (**a**) *Acaulospora*, (**b**) *Gigaspora*, (**c**) *Glomus*, (**d**) *Orbispora*, (**e**) *Scutellospora*, (**f**) Diversisporales, (**g**) Gigasporales, (**h**) Glomerales. Bars followed by the same letter do not differ statistically.

A difference in the abundance of orders was observed across the gradient, Gigasporales and Glomerales were more abundant in LCC, and Diversisporales predominated in LCC and MCC (Figure 2). The order Gigasporales was an indicator of the LCC and MCC areas, with the families Gigasporaceae and Scutellosporaceae also being indicators of the same areas (Table 3). Regarding genera, it was observed that *Scutellospora* was indicative of the LCC area, *Racocetra* for the MCC area, and *Gigaspora* and *Orbispora* were indicators of the LCC and MCC areas. The density of the genera differed according to the clay gradient, with *Glomus* and *Scutellospora* significantly more abundant in LCC than in the other areas, while *Acaulospora*, *Gigaspora* and *Orbispora* were predominant in LCC and MCC (Figure 2). Despite these variations, there was no difference in the diversity of AMF among environments (F = 1.82, *p* > 0.05).

The five most abundant species (*Glomus macrocarpum*, *G. brohultii*, *G. glomerulatum*, *Glomus* sp. 4 and *Glomus* sp. 3) together represented 87.32% of all spores identified. In addition, these species were also considered dominant in all areas, a parameter which also included *Funneliformis halonatus*. On the other hand, dominant species represent 30% of the species in LCC, 22% in MCC and 19% of the taxa in HCC.

Nine AMF species were found exclusively in LCC (*Acaulospora* sp. 4, *Dentiscutata scutata*, *Gigaspora* sp., *Paraglomus pernambucanum*, *Rhizoglomus intraradices*, *Sclerocarpum* sp., *Scutellospora alterata*, *Scutellospora* sp. and *Tricispora* sp. 1), 13 were unique to MCC (*Acaulospora denticulata Acaulospora herrerae, Acaulospora* sp. 6, *Acaulospora* sp. 7, *Archaeospora* sp., *Entrophospora* sp. 1, *Entrophospora* sp. 2, *Paraglomus occultum, Racocetra beninensis, Racocetra fulgida, Racocetra persica, Racocetra verrucosa and Tricispora* sp. 2), and 11 were recorded only in HCC (*Acaulospora* sp. 8, *Ambispora* sp., *Bulbospora minima*, *Cetraspora* sp., *Cetraspora pellucida*, *Funneliformis mosseae*, *Intraornatospora intraornata*, *Glomus* sp. 10, *Glomus* sp. 9, *Paradentiscutata maritima* and *Paradentiscutata* sp.), only 26 species were common to all three areas. The Sørensen similarity index showed that the AMF assembly between LCC and HCC has 61% similarity, between HCC and MCC the composition was about 60% similar, and LCC and MCC showed 74% similarity of species of these fungi.

Seven species were considered indicators for at least one area: *Acaulospora* sp. 5, *Glomus* sp. 9, *Glomus* sp. 10 and *Cetraspora pellucida* for HCC; *Acaulospora* sp. 6 for MCC; *Scutellospora tepuiensis* and *Acaulospora* sp. 4 for LCC (Table 3). Three species (*Gigaspora margarita, Orbispora pernambucana* and *Scutellospora tepuiensis*) were indicators of the LCC and MCC areas.

**Figure 3:**
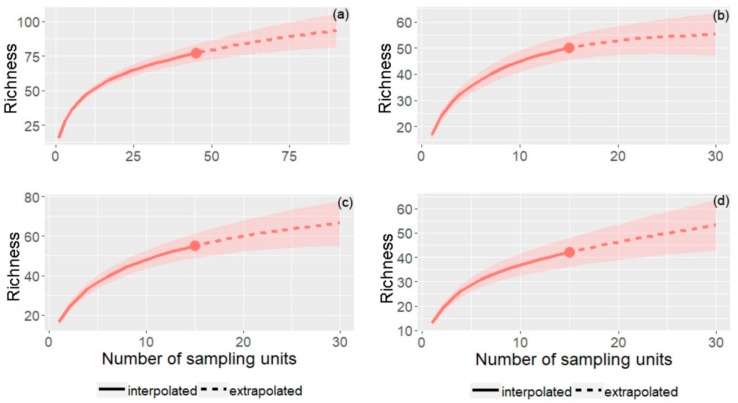
Species accumulation curve of AMF species across a clay gradient (LCC: Low Clay Content, MCC: Medium Clay Content, HCC: High Clay Content). (**a**) Total Richness, (**b**) LCC, (**c**) MCC, (**d**) HCC.

According to the Jackknife 1 richness estimator, it was possible to recover 77% of the total AMF richness expected for the area (Figure 3). In relation to each environment, the species accumulation curves showed that the LCC area reached 82% of the estimated potential for AMF richness, while 77% and 76% of the expected number of taxa were obtained for MCC and HCC, respectively (Figure 3).

### 2.3. AMF Richness and Assembly Composition in the Trap Cultures

A total of 38 AMF species were identified in the trap cultures, belonging to 16 genera, with *Acaulospora* and *Glomus* being the most representative, having eleven and ten species respectively. Twelve AMF species were shared among the trap cultures of the three areas, five were exclusive in the LCC culture, six in MCC and four in HCC. From the trap cultures it was possible to include three species in the list (*Claroideoglomus claroideum, Rhizoglomus custos* and *Sacculospora baltica*), bringing the total number of taxa identified to 80 (Table 4). These three species were recorded exclusively in trap cultures and were not found in the field; 42 AMF taxa were recorded only in the field, and 35 were present in both the field and in the trap cultures.

### 2.4. Composition of the AMF Assembly from Field Samples

Permanova showed that there was a significant difference in the composition of the AMF assembly only between the HCC and LCC areas (F = 3.7, *p* < 0.001). Comparison between field and trap culture samples showed that the field assembly of AMF species has a different composition from that of trap cultures (F = 15.6, *p* < 0.001).

**Figure 4:**
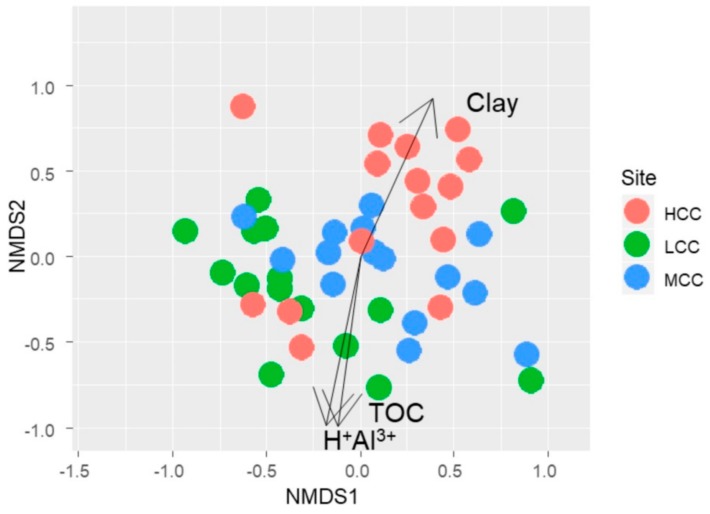
Analysis of multidimensional scaling of the AMF community correlated with soil data in three areas with a clay gradient (LCC: Low Clay Content, MCC: Medium Clay Content, HCC: High Clay Content). Stress value: 0.16.

Multidimensional non-metric scaling analysis (NMDS) showed that soil attributes were correlated with the composition of the AMF assembly in the study area (Figure 4). Clay was related to the AMF assembly of the HCC area (R^2^ = 0.24; *p* < 0.05), while TOC (R^2^ = 0.22; *p* < 0.05) and H^+^Al^3+^ (R^2^ = 0.18; *p* < 0.05) were structurers of the AMF assembly of the LCC area (*p* < 0.05).

## 3. Discussion

A difference was observed between the AMF assembly in the area with lower and higher clay contents (LCC and HCC). Soil is formed of particles of different sizes, constituting aggregates with varied porosity, where gas exchange, water and nutrient distribution occur, not to mention the presence of microorganisms and root expansion [23]. Thus, the physical properties of the soil can modify the dynamics of AMF species [19]. Sandy soils are lighter and have larger particles, while clayey soils are dense and composed of fine particles that limit root expansion and possibly also the distribution of mycelium [24]. Additionally, sandy soils have larger porous spaces that can facilitate the metabolic processes of microorganisms [25], providing better conditions for exchange between the soil and roots by the AMF assembly in LCC.

Soil texture has commonly been related to changes in the AMF communities [18,24,26] and also in changes in plant communities [27,28]. In the present study, a higher content of clay affected the AMF assembly in HCC, leading to a decrease in the AMF richness. In semi-arid forests in Brazil, clay was shown to be the main influencer of AMF richness [29], and was also negatively related to AMF diversity in ecosystems in a semiarid region of China [18]. The higher clay contents and the revealed shift in the AMF communities certainly is also related in our study to the shift of the plant communities from shrublands in LCC towards more arboreal habitats in MCC and HCC.

There was a marked difference in the distribution of AMF species along the gradient, especially in relation to the presence of species of various genera of Gigasporales (*Gigaspora*, *Scutellospora*, *Orbispora*, *Dentiscutata*, *Racocetra* and *Gigaspora*) that were not identified in the area with highest clay content (HCC). In general, representatives of Gigasporales were indicators of the sandiest area (LCC) or of LCC and MCC. Taxa of this order have a preference for sandier soils, as has already been observed in other studies [8,30], because they may be able to compete in these environments [19]. Members of the family Gigasporaceae are known for their competitive life strategy through increased hyphal production, increased carbon demand, and increased phosphorus absorption from the soil, characteristics which are important in environments with low levels of soil nutrients [31,32]. Gigasporoid species produce a large amount of extraradicular mycelia, with a higher proportion of propagules in the soil in relation to the root interior [33]. This functional characteristic is an important strategy in soils with limiting conditions such as low nutrient content [31], as found in rocky outcrops like in LCC, where nutrients are scarce, coming directly from the rock matrix and the decomposition of accumulated organic plant matter. Greater mycelial growth provides greater opportunities for soil exploration, ensuring a better nutritional contribution to the hosts. In addition, through the increased production of hyphae, the representatives of this order possibly assist in the stabilization of the substrate, and contributes to the increase of soil aggregation.

AMF families may occupy specific niches according to preferences in biotic and abiotic properties, for example, Gigasporaceae has been found to be sensitive to precipitation, showing that soil moisture may also have been determinant in this study [34]. Humidity modifies the AMF community, because soil water can be determinant in the metabolic processes of fungi and indirectly affect the distribution of AMF species [18]. In general, all areas presented greater abundance of representatives of the Glomeraceae family, who have different propagation strategies [19], providing greater adaptation to various soil situations. Regarding the richness of genera in the study site, there was a predominance of *Acaulospora*, which has greater capacity to withstand environmental stresses such as soil acidity [31]. In a modeling analysis of AMF families, Veresoglou et al. [34] found that Acaulosporaceae was related to more acidic soils, corroborating the results obtained in this study (pH 4.9–5.3), showing greater capacity of species of this family to survive in restrictive environments.

As there were differences in the AMF assembly only between the LCC and HCC areas, other factors may be associated with the distribution of AMF species. In the study area, vegetation may also have been one of the modelers of the mycorrhizal fungus assembly, which may respond in different ways to changes in vegetation [7]. In this sense, the vegetation in LCC presents a shrubby physiognomy, while in HCC the physiognomy is arboreal, presenting marked differences in the vegetational types; on the other hand, the MCC area represents an intermediate appearance, including both shrubs and trees. Differences in soil texture may have contributed to plant species distribution, indirectly influencing AMF assemblage. Plants obtain nutrients from the soil according to their needs, and thus respond to the AMF assemblies in different ways, selecting the AMF directly through their influence on the soil, or indirectly through internal pressures on the root [9]. Thus, the AMF community may be related to that of the plants along soil gradients [35].

In this study, a high number of AMF species was found (80) when compared to other studies in semi-arid regions with montane forests in Brazil, where 50 and 70 species were recorded [30,36]. Although the richness was high, only five species constitute the majority of the spores (ca. 87%) in the study site. The predominance of just a few AMF species in these areas may be explained by the ability of some AMF to form easily a mycorrhizal association with virtually most plant species. On the other hand, subdominant species, and species with low abundances to rare occurrences, might be more sensitive to soil texture and vegetation type, or other environmental stressors. Host preference was several times reported from different environments [37,38,39]. On the other hand, trap culture studies with single host plant species revealed that the large majority of AM fungi, present in the soils, could be propagated on single plant species [40,41]. In this respect, it is also notable that, even for specific plant species, differences between intraradical and extraradical AMF communities might exist, although most AMF species prefer to explore the soils and thus, preferentially live and sporulate in the rhizospheric soil environment. Intraradical and extraradical sporulators are all detected with our isolation techniques, as long as they sporulate within the rhizospheric soils or within the roots. The higher spore density in the LCC did not reflect a difference in the diversity of AMF in this area; however, there was a predominance of some genera and types of spore formation in the LCC, which are possibly associated with lower clay content, because high levels of clay can reduce spore formation [19]. The small pores of clay soil can impair root growth and even cause rupture in the cortex cells, reducing the colonization points for AMF [22]. In addition, a reduction in soil nutrient content can promote an increase of spore production in the field [42].

The indicator species represent species adapted to the specific conditions of the areas, such as *Scutellospora tepuiensis*, described in mountainous plateaus in Venezuela (South America) with acidic (pH 5.7–5.9) and sandy soils and shrubby vegetation [43], characteristics similar to those found in LCC. Gigasporoid species indicative of some areas, such as *Cetraspora pellucida*, *Gigaspora margarita* and *S. calospora*, are found in sand dune ecosystems around the world, and are thus considered cosmopolitan [44]. *C. pellucida* was an exception among gigasporoid species, being an indicator of HCC. This species has also been found in several ecosystems, has a wide geographic distribution in Brazil [45], and can adapt to various conditions, including clay soils. The LCC and MCC areas presented common indicator taxa, and had a higher percentage of similarity of AMF species, which can be justified by the similarities in soil properties between these areas.

There was a difference in the AMF assembly in the field compared to that observed in the trap cultures. The trap culture represents a microenvironment that provides different conditions and hosts from those found in the field [46], subjecting the AMF to reduced space, in which the taxa are submitted to greater competition. Thus, the AMF can assume other survival strategies and form different types of propagules. About 45% of the species identified in the field were recovered in the trap cultures, thus a significantly smaller quantity of taxa in this bioassay. This is possibly occurring due to the different substrate, to the disturbance in the formation of the substrate, interfering in the dynamics of the species, or the duration of the trap culturing [47,48]. The cultures were assembled with dilution in sand. This modification of the substrate may also have contributed to greater aeration of the soil, which may have provided different assemblies of AMF from the soil collected from the field. Species found exclusively in the trap culture suggest that some AMF taxa were under other forms of propagules in the field and sporulated under culture conditions [49].

We found lower AMF richness in the area with higher clay content, corroborating the hypothesis of the study. Changes in soil texture can be determinant in the distribution of AMF species and restrict the growth of propagules, for example, Gigasporaceae species have thicker hyphae than other AMF families [33], and consequently spread less in clayey soils. Although Gigasporaceae is predominant in sandy soils with low humidity and low organic matter content [19], in this work, the sandy soil had a higher content of TOC and moisture, because of the local environmental properties: LCC is a shrubby area with rocky outcrops (where organic matter is deposited in the small space occupied by the plant) at the upper top of a mountainous area, where higher rainfall occurs possibly resulting in a loss of clay particles by soil erosion. Nevertheless, these conditions did not prevent a greater predominance of the family Gigasporaceae, since sand appears to be the main driver favoring this AMF group.

## 4. Materials and Methods

### 4.1. Study Area

The study area is located in the semi-arid region of the Brazilian Northeast, where vegetation of tropical dry forests predominates, called Caatinga [50]. These ecosystems are highly diversified and composed of different physiognomic features, forming a vegetative mosaic of dry forests, shrubby, humid montane forests, and cerrado (the Brazilian savanna).

The collection site selected was Serra da Boa Vista which is located in the municipality of Brejo da Madre de Deus, state of Pernambuco, and the peak of Boa Vista is the highest point in the state at an altitude of 1195 m. The city’s climate is tropical with autumn-winter rains, type As’ according to the Köppen classification, with an average annual temperature of 22 °C and average annual precipitation of 844 mm [51]. The soil of the area was formed in the Proterozoic period, being composed of granite and diorite [52].

**Figure 5:**
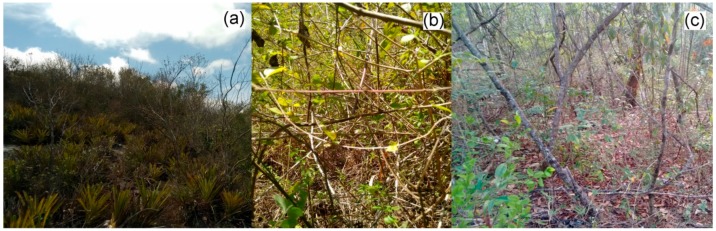
Collection sites with a clay gradient (**a**) LCC, (**b**) MCC, and (**c**) HCC.

Three areas were chosen following an increasing gradient in clay content: low clay content (LCC), medium clay content (MCC) and high clay content (HCC). These areas present differences in the plant community (Figure 5): in LCC, there is shrubby vegetation along elevated rocky outcrops, similar to cerradão, a type of cerrado ecosystem (Table 5). Humid plateau areas exist in the middle of the caatinga where species of cerrado and rupestrian fields are commonly found, forming peculiar environments with endemic representatives [53]. On the other hand, HCC has arboreal vegetation with a transition physiognomy between the semi-arid biome (Caatinga) and the montane rainforest vegetation. Some of the species are shared between these environments, because the transition area occurs gradually, according to changes in altitude and topography of the terrain [54] (Table 5). MCC is also considered a transition area between a dry and humid environment, but this environment was more degraded, at the time of collection, this could be observed by agricultural activities and the dominance of forage grasses in large clearings (Table 5).

### 4.2. Collection

Collection was carried out in November 2016 in the dry period. In each area, three 30 × 30 m plots were delineated, with a minimum distance of 50 m among them. In each plot, five soil samples were collected, constituting 15 samples per area. In total, 45 soil samples were collected from 0–20 cm depth close to the rhizosphere of plants. The samples were stored in plastic bags and taken to the Mycorrhizal Laboratory at UFPE for the evaluation of the AMF assembly. The samples were kept at room temperature (about 25 ° C) to reduce soil moisture and prevent spore germination.

### 4.3. Trap Culture

Part of the soil was used in trap cultures to obtain viable spores for morphological identification, aiming to better represent the AMF assembly. Well established mycotrophic plants were used to allow field species to colonize and sporulate. Trap cultures were set up one month after collection. From each sample, 500 g of soil were separated and mixed with 500 g of washed sand. The soil of the field was diluted with sand to create a more aerated and nutrient-poor substrate, providing optimal conditions for colonization and sporulation of the species. The substrate was deposited in two-liter plastic pots, where corn and sorghum were sown and remained in a greenhouse for eight months, being watered daily. After this period, the plants were subjected to hydric stress for 20 days in order to favor AMF sporulation. Subsequently, 50 g of the soil were removed for species evaluation.

### 4.4. Spore Extraction and Morphological Identification

The spores were extracted from 50 g of soil from the field and trap culture samples using the wet sieving method [55], followed by centrifugation in water and sucrose (50%). The spores were separated and mounted according to morphotypes on slides with PVLG (polyvinyl alcohol lactoglycerol) and PVLG + Melzer reagent (1:1) and were analyzed using a light microscope (40×). The species were identified using identification manuals [56] and recent descriptions of species. The spores of each species were counted and the sporocarps were considered as one unit.

### 4.5. Soil Analyses

For the chemical analysis, soil samples were air-dried, crumbled and passed through 2-mm mesh sieves. The pH and electrical conductivity of the soil were determined in a soil:water solution (1:2.5). Potential acidity (H^+^Al^3+^) was measured after extraction with calcium acetate. Phosphorus, sodium and potassium (P, Na and K^+^) were extracted with Mehlich I solution, with P quantified by spectrophotometry and Na and K^+^ by flame photometry [57]. Aluminum, calcium and magnesium (Al^3+^, Ca^2+^ and Mg^2+^) were extracted with potassium chloride (KCl 1 M). Al^3+^ was quantified by titration and Ca^2+^ and Mg^2+^ were quantified by atomic absorption spectrometry [57]. Total organic carbon (TOC) was measured by wet oxidation of carbon (Walkley-Black method), according to Tedesco et al. [58]. Particle size analyses were performed by the pipette method, and the humidity was determined after drying the soil in an oven at 105 °C for 24 h.

### 4.6. Statistical Analyses

The ecological measures of species richness per sample, relative abundance (RA), frequency of occurrence (FO), the Shannon-Weiner diversity (H) and Sørensen indices were used to evaluate the distribution of AMF species. Relative abundance was determined as the ratio of the number of spores of each species to the total number of spores. The frequency of occurrence (FO) of the species was estimated according to the equation: FO = Ji/k where, FO = frequency of occurrence of the species, Ji = number of samples where the species occurred in the analyzed area, k = total number of soil samples. The species were classified as dominant (FO > 50%), very common (31% ≤ FO ≤ 50%), common (10% ≤ FO ≤ 30%) or rare (FO < 10%) [59]. The Shannon species diversity index was calculated according to the equation H’ = −Σ(Xi/Xo) × log(Xi/Xo), where Xi is the number of spores of each species; Xo is the total number of spores of all species. To evaluate the similarity of the AMF species among areas, the Sørensen index [60] was used. To estimate the number of species found in each area, the first-order Jackknife index (Jackknife 1) was determined.

Indicator species analysis [61] was applied to species, genus, family, order and type of AMF spore formation for each area. The analysis also considered whether any taxa would be indicative of a combination of two or more sites. The indicator value (IndVal) was calculated and the significance of the values was obtained by the Monte Carlo test, with each of the categories analyzed; taxa were considered indicators when they presented *p* < 0.05 and IndVal greater than or equal to 25%.

The distribution of the AMF species among the environments was observed through NMDS (non-metric multidimensional scaling) using data of relative abundance of each AMF species and the Sørensen index. The physical and chemical properties of the soil were used to verify the correlation of these factors with the ordination axes through Envfit analysis. Multivariate permutation analysis (PERMANOVA) was performed to compare community composition across the clay gradient using relative abundance. The species presence-absence data were considered to verify, whether differences between trap and field cultures in the AMF species composition existed.

The Shannon index, richness, spore density overall and by genus, family, order and type of spore formation, as well as soil attributes were submitted to normality and heterogeneity tests. The Shannon index and richness met these tests and were then submitted to analysis of variance and the means compared by the Tukey test (*p* ≤ 0.05). As the other analyses did not meet these requirements, the non-parametric Kruskal-Wallis test was performed (*p* ≤ 0.05). All analyses and figures were developed using RStudio 3.5.1 software [62], using the following functions and packages: for the species accumulation curves and richness estimator the functions ‘specpool’ and ‘specaccum’ of the vegan package [63] and ‘iNEXT’ of the iNEXT package [64] were used; the indicator species analysis was done using the function ‘multipatt’ of the indicspecies package [65]; the NMDS, ENVFIT and PERMANOVA were done using the functions ‘metaMDS’, ‘envfit’ and ‘adonis’ of the vegan package [63].

## 5. Conclusions

The distribution of AMF species was affected by soil texture and vegetation type, with differences in predominance among families, genera and species in relation to the clay gradient. Clay, TOC and potential acidity were the main soil factors related to the AMF assembly in the environment studied. The area with the highest clay content had reduced AMF richness, indicating that this texture may be more restrictive for AMF species. On the other hand, the area with the lowest clay content concentrated most types of spore formation and certain orders of AMF, showing that in this area the conditions were favorable for the propagation of AMF spores. Representatives of the order Gigasporales are sensitive to changes in soil texture, presenting a reduction in the abundance of species of this order as the clay content in the soil increases, indicating the functional role of the species of this AMF group. In general, the composition of the AMF assembly differed among areas showing that especially subdominant and more rare AMF species were sensitive to the environmental changes. This study clearly showed that large differences in AMF communities can occur even between short distances in one single landscape, here represented by different habitats of a relatively humid mountain top within the semi-arid NE Brazil. The importance of different vegetation types for the establishment of AMF communities has to be further explored. However, the AM fungi are already expected to gain increasing significance for efficient vegetation restoration of degraded areas and protection of diverse savanna and forest environments, which are increasingly threatened by the changing climates and increased soil erosion.

## Figures and Tables

**Table 1:** Physical attributes of soil in three areas with a clay gradient.

Site	(%)		
	Sand	Silt	Clay
LCC—Low Clay Content	55.91a	28.89a	15.20c
MCC—Medium Clay Content	47.68b	27.16a	25.16b
HCC—High Clay Content	45.12b	21.48b	33.40a

Averages followed by the same letter in the same column do not differ statistically.

**Table 2:** Soil chemical attributes in three areas with a clay gradient (LCC: Low Clay Content, MCC: Medium Clay Content, HCC: High Clay Content). TOC: total organic carbon, EC: electrical conductivity, H^+^Al^3+^: potential acidity.

	(Water)	mg dm^−3^		Cmol_c_ dm^−3^					dS m^−1^	g dm^−3^
Site	pH	P	K	Na	Mg	Ca	H^+^Al^3+^	Al	EC	TOC
LCC	4.90b	5.72ab	0.28b	0.14a	5.42b	0.35b	5.17a	2.09a	0.15b	27.58a
MCC	5.31a	5.24b	0.59a	0.09a	6.57ab	0.84a	4.29a	0.75b	0.16b	24.61a
HCC	5.31a	6.49a	0.70a	0.08a	7.73a	0.70a	2.87b	0.43b	0.19a	20.24b

Averages followed by the same letter in the same column do not differ statistically.

**Table 3:** Type of spore formation, species, genus, family, order of AMF indicators of some area according to the Monte Carlo test (IV ≥ 25%, *p* < 0.05). LCC: Low Clay Content, MCC: Medium Clay Content, HCC: High Clay Content.

Heading	Site	IV	P
Order			
*Gigasporales*	LCC + MCC	96.6	0.001
Family			
*Gigasporaceae*	LCC + MCC	87.6	0.001
*Scutellosporaceae*	LCC + MCC	81.2	0.001
Genus			
*Scutellospora*	LCC	78.4	0.002
*Racocetra*	MCC	51.6	0.03
*Gigaspora*	LCC + MCC	87.6	0.001
*Orbispora*	LCC + MCC	68.3	0.008
Species			
*Acaulospora* sp. 4	LCC	57.7	0.008
*Scutellospora tepuiensis*	LCC	85.3	0.001
*Acaulospora* sp. 6	MCC	51.6	0.034
*Acaulospora* sp. 5	HCC	59.0	0.04
*Cetraspora pellucida*	HCC	51.6	0.025
*Glomus* sp. 9	HCC	63.2	0.001
*Glomus* sp. 10	HCC	51.6	0.022
*Gigaspora margarita*	LCC + MCC	81.6	0.001
*Orbispora pernambucana*	LCC + MCC	68.3	0.009
*Scutellospora calospora*	LCC + MCC	68.3	0.007
Type of spore formation			
Gigasporoid	LCC + MCC	96.6	0.001

**Table 4:** Relative abundance (RA) and frequency of occurrence (FO) of AMF species in the field and soil trap cultures in three areas with a clay gradient (LCC: Low Clay Content, MCC: Medium Clay Content, HCC: High Clay Content).

Species	Field							Trap Culture	
	LCC	MCC	HCC	LCC	MCC	HCC	LCC	MCC	HCC
	RA	RA	RA	FO	FO	FO	RA	RA	RA
*Acaulospora denticulata*		0.01			13.33				
*Acaulospora foveata*	0.02	0.05	0.03	26.67	20.00	26.67		0.04	0.05
*Acaulospora herrerae*		0.003			6.67				
*Acaulospora lacunosa*	0.01	0.003	0.01	13.33	46.67	6.67			
*Acaulospora longula*	0.26	0.03	0.03	60	46.67	26.67	0.31		0.07
*Acaulospora mellea*	0.50	0.41	0.07	66.67	46.67	66.67	0.5	0.03	0.14
*Acaulospora morrowiae*	0.14	0.18	0.33	40	53.33	73.33			0.19
*Acaulospora rehmii*	0.01	0.02		6.67	20			0.05	
*Acaulospora scrobiculata*	0.65	0.08	0.05	60	53.33	26.67	0.12	0.04	0.04
*Acaulospora spinosa*	0.013	0.08	0.02	13.33	33.33	20			
*Acaulospora spinosissima*	0.01	0.003	0.01	13.33	6.67	13.33	0.03	0.03	
*Acaulospora spinulifera*	0.25	0.05		20	20				
*Acaulospora* sp. 1	0.01	0.04		13.33	13.33		0.01		
*Acaulospora* sp. 2	0.003	0.003		6.67	6.67		0.03		0.01
*Acaulospora* sp. 3	0.07	0.02	0.01	13.33	33.33	20	0.01	0.01	0.03
*Acaulospora* sp. 4	0.45			33.33					
*Acaulospora* sp. 5	0.03	0.02	0.14	20	6.67	46.67			
*Acaulospora* sp. 6		0.08			26.67			0.01	0.01
*Acaulospora* sp. 7		0.01			6.67				
*Acaulospora* sp. 8			0.003			6.67			
*Ambispora appendicula*	0.04	0.02	0.02	33.33	20	26.67	0.80	0.62	0.16
*Ambispora* sp.			0.07			6.67			
*Archaeospora* sp.		0.003			6.67				
*Bulbospora minima*			0.12			20		0.01	
*Cetraspora gilmorei*	0.01	0.03	0.003	13.33	26.67	6.67		0.01	
*Cetraspora pellucida*			0.02			26.67			
*Cetraspora* sp.			0.003			6.67			
*Claroideoglomus claroideum*									0.01
*Claroideoglomus etunicatum*	0.08	0.01	0.01	13.33	13.33	6.67	0.04	0.03	0.01
*Dentiscutata cerradensis*	0.02	0.02		33.33	20		0.03		
*Dentiscutata scutata*	0.01			6.67					
*Dominikia aurea*	0.14	0.02	0.01	13.33	6.67	6.67	0.91		0.12
*Dominikia bernensis*	0.51		0.03	6.67		6.67			
*Entrophospora* sp. 1		0.01			13.33				
*Entrophospora* sp. 2		0.003			6.67				
*Funneliformis halonatus*	0.15	0.21	0.26	60	93.33	60	0.32	0.22	0.38
*Funneliformis mosseae*			0.003			6.67			0.01
*Fuscutata savannicola*	0.01	0.01	0.01	13.33	13.33	20			
*Gigaspora decipiens*	0.02	0.01		20	13.33				
*Gigaspora gigantea*	0.04	0.02		26.67	20				
*Gigaspora margarita*	0.40	0.11		66.67	66.67		0.07	0.05	
*Gigaspora* sp.	0.01			6.67			0.08		
*Glomus brohultii*	16.79	4.20	3.47	100	100	100	7.86	5.15	9.03
*Glomus glomerulatum*	16.47	4.62	3.62	100	100	100	4.65	2.14	2.08
*Glomus macrocarpum*	8.39	5.25	3.71	100	100	100	15.23	10.78	5.36
*Glomus microcarpum*	0.37	0.12	0.07	66.67	73.33	46.67	0.04		0.05
*Glomus* sp. 1	0.03	0.02		33.33	26.67				
*Glomus* sp. 2	0.64	0.19	0.03	40	73.33	33.33	0.22	0.03	
*Glomus* sp. 3	1.21	1.07	1.00	86.67	100	100	0.45	0.5	0.65
*Glomus* sp. 4	4.92	4.25	8.37	86.67	100	100	3.16	9.57	12.97
*Glomus* sp. 5	0.38	0.25	0.42	66.67	53.33	26.67	0.62	0.88	1.66
*Glomus* sp. 6	0.22	0.003		6.67	6.67		0.05		
*Glomus* sp. 7	0.01	0.003	0.01	13.33	6.67	26.67			
*Glomus* sp. 8		0.53	0.11		26.67	26.67			
*Glomus* sp. 9			0.46			40			0.59
*Glomus* sp. 10			0.16			26.67			
*Intraornatospora intraornata*			0.01			13.33			
*Paradentiscutata bahiana*	0.03		0.02	13.33		6.67			
*Paradentiscutata maritima*			0.01			6.67			
*Paradentiscutata* sp.			0.01			6.67			
*Paraglomus occultum*		0.003			6.67				
*Paraglomus pernambucanum*	0.003			6.67					
*Orbispora pernambucana*	0.43	0.14		53.33	40		0.01	0.03	
*Racocetra beninensis*		0.01			6.67				
*Racocetra fulgida*		0.003			6.67				
*Racocetra persica*		0.01			6.67				
*Racocetra verrucosa*		0.003			6.67			0.03	
*Rhizoglomus clarum*	0.01	0.003	0.003	20	6.67	6.67			
*Rhizoglomus custos*								0.18	
*Rhizoglomus intraradices*	0.01			6.67					
*Sacculospora baltica*								0.26	
*Sclerocarpum* sp.	0.01			6.67					
*Sclerocystis clavispora*		0.02	0.01		20	13.33			
*Scutellospora alterata*	0.003			6.67					
*Scutellospora calospora*	0.25	0.29		60	33.33				
*Scutellospora tepuiensis*	0.73	0.01		73.33	13.33		0.07		
*Scutellospora* sp.	0.003			6.67					
*Septoglomus constrictum*		0.003	0.003		6.67	6.67		0.03	0.01
*Tricispora* sp. 1	0.01			6.67					
*Tricispora* sp. 2		0.01			13.33				
Total	50	55	42				25	25	23

RA: relative abundance, FO: frequency of occurrence.

**Table 5:** Geographical coordinates, altitude and predominant phytophysiognomy in three areas with a clay gradient (LCC: Low Clay Content, MCC: Medium Clay Content, HCC: High Clay Content).

Site	Latitude	Longitude	Altitude	Phytophysiognomy
LCC	8°9′24.65″ S	36°23′36.05″ W	1170	Shrubby
MCC	8°9′47.31″ S	36°23′14.36″ W	1030	Shrubby-arboreal
HCC	8°10′43.53″ S	36°23′25.61″ W	900	Arboreal
